# A detailed nutritional assessment may further elucidate the relationship between vitamin D and thyroid function

**DOI:** 10.1590/1806-9282.20250707

**Published:** 2025-10-17

**Authors:** Jun-Xiang Zhao, Ya-Qi Chen, Lian-Ping He, Ying-Rui Huang

**Affiliations:** 1 Taizhou University, School of Medicine – Jiaojiang, China.

Dear Editor,

We read with great interest the article by Almeida et al.^1^ titled "Vitamin D and thyroid function of pregnant women in a sunny region: is there any connection?" published in your esteemed journal. The study's exploration of the relationship between vitamin D and thyroid function in pregnant women in Northeast Brazil offers valuable insights, yet it also prompts several considerations.

The research's cross-sectional design, despite its limitations, provides a snapshot of the relationship in a specific population. The exclusion of women with pre-existing thyroid disorders and those on relevant supplements is a commendable approach, as it helps isolate the variables of interest. However, the relatively small sample size and single-center setting might limit the generalizability of the findings. A larger, multi-center study could have provided more robust results applicable to a broader range of pregnant women.

The study's finding of a low prevalence of vitamin D deficiency (3.9%) contrasts with reports from other regions, highlighting the influence of geographical and environmental factors. Nevertheless, the high rate of vitamin D insufficiency (81%) indicates a significant concern that warrants attention. The lack of a significant correlation between vitamin D status and thyroid hormones is intriguing, especially when compared to previous studies with differing results^2^. This disparity emphasizes the need for further research to understand the complex interplay between these factors.

In the discussion section, the authors appropriately acknowledge the potential confounding factors such as ethnicity, sunlight exposure, and dietary habits. It is well established that vitamin D levels are strongly associated with various health conditions and are also influenced by various factors^3-8^. However, a more in-depth analysis of these factors could have been conducted. For example, given the importance of diet in vitamin D and iodine intake, a detailed dietary assessment might have provided additional insights into the relationship between vitamin D status and thyroid function.

Regarding the classification of vitamin D status, the ongoing debate about what constitutes insufficiency is well presented. It would be beneficial if future research could focus on establishing more accurate and region-specific thresholds for vitamin D sufficiency during pregnancy. This would greatly assist in formulating more effective public health strategies.

Finally, considering the implications for prenatal care, the high prevalence of vitamin D insufficiency calls for routine screening and appropriate supplementation. The Brazilian Ministry of Health's policy on vitamin D supplementation during pregnancy is a positive step, but more research is needed to optimize the dosage and delivery of such supplements.

We believe that the findings of this study contribute to the growing body of knowledge on maternal–fetal health. However, further research in this area is essential to clarify the relationship between vitamin D and thyroid function in pregnant women and to improve pregnancy outcomes. We look forward to seeing more studies in this field that build on these findings.

## Data Availability

The datasets generated and/or analyzed during the current study are available from the corresponding author upon reasonable request.
